# A study on the mathematical learning knowledge structures of junior high school mathematically gifted students in Nanning, China

**DOI:** 10.3389/fpsyg.2026.1837988

**Published:** 2026-07-22

**Authors:** Wei Zhou, Cheng Huang, Rongrong Huang

**Affiliations:** 1 Institute of Future Education, Nanning Normal University, Nanning, China; 2 College of Civil Aviation Transportation, Chengdu Aeronautic Polytechnic University, Chengdu, China

**Keywords:** gender differences, learning knowledge, mathematical knowledge, mathematical learning knowledge structure, mathematically gifted students, mathematics achievement, mathematics-specific learning knowledge

## Abstract

**Introduction:**

This study investigated the mathematical learning knowledge structures of junior high school mathematically gifted students in Nanning, China, and examined gender differences and the relationship between these knowledge structures and mathematics achievement.

**Methods:**

A quantitative cross-sectional survey design was adopted. The participants were 90 Grade 9 mathematically gifted students selected through criterion-based purposive sampling from nine classes in a junior high school. Data were collected using an adapted Mathematical Learning Knowledge Questionnaire and a researcher-designed Mathematics Achievement Test. The instruments were reviewed by three experts for content validity. Reliability analysis showed acceptable to high internal consistency, with Cronbach’s alpha values of 0.948 for the overall questionnaire, 0.866 for MK, 0.862 for LK, 0.886 for MLK, and 0.827 for the mathematics test.

**Results:**

Independent-samples *t*-tests revealed no statistically significant gender differences in MK, LK, MLK, or the overall mathematical learning knowledge structure. Multiple regression analysis showed that the model significantly predicted mathematics achievement, *F*(4, 85) = 33.598, *p* < 0.001, *R*^2^ = 0.613. LK and MLK were significant positive predictors, whereas gender and MK were not significant unique predictors.

**Discussion:**

The findings suggest that mathematics achievement among mathematically gifted students is more closely related to learning knowledge and mathematics-specific learning knowledge than to gender or mathematical knowledge alone. Teachers and curriculum planners should give greater attention to students’ learning strategies, self-regulation, and mathematics-specific learning processes.

## Introduction

Mathematically gifted students are often expected to demonstrate high levels of mathematics achievement because they usually show strong interest in mathematics, rapid understanding of mathematical relationships, flexible reasoning, and advanced problem-solving ability. However, mathematical giftedness does not automatically lead to consistently high mathematics achievement. Some mathematically gifted students may have strong mathematical potential but still differ in how they organize mathematical knowledge, regulate their learning, apply learning strategies, and transform mathematical understanding into actual performance. Therefore, the education of mathematically gifted students should not focus only on the amount of mathematical knowledge they possess, but also on how they learn mathematics and how they connect mathematical knowledge with learning processes.

Previous studies have provided important theoretical explanations of giftedness and mathematical giftedness. Gardner (1999) and Renzulli (1986) emphasized that giftedness involves multiple abilities and potentials, including intellectual ability, creativity, motivation, and social and self-related characteristics. In the field of mathematics, Krutetskii (1976) suggested that mathematically gifted students often perceive the world through a mathematical perspective and are able to generalize mathematical relationships and operations quickly. Similarly, Singer et al. (2016) noted that mathematically gifted students may demonstrate advanced mathematical reasoning, flexible mental processes, and distinctive approaches to mathematical problem solving. Moreover, previous studies have described mathematically gifted students as learners who may complete mathematical tasks with unusual speed and accuracy, engage in mathematical thinking that differs qualitatively from that of their peers, identify relationships among mathematical concepts without direct instruction, and sometimes skip intermediate steps in problem solving (Koshy et al., 2009; Leikin, 2010; Leikin and Lev, 2012; Reed, 2004). These characteristics further suggest that mathematical giftedness should be understood not only in terms of high performance, but also in terms of distinctive mathematical thinking and learning processes. However, mathematical giftedness and mathematics achievement should not be regarded as identical. A student may possess mathematical giftedness without always achieving high mathematics scores, and high achievement in mathematics does not necessarily indicate mathematical giftedness.

Recent research has further emphasized that mathematical giftedness is a multidimensional construct rather than a single form of high mathematical ability. Sipahi and Bahar (2024), in a systematic review of empirical studies on the cognitive characteristics of mathematically gifted individuals, reported that mathematical giftedness involves both domain-specific abilities, such as mathematical reasoning, problem solving, and mathematical creativity, and domain-general abilities, such as memory, reasoning, and visual-spatial ability. Özdemir et al. (2024) also showed that research on mathematical giftedness has developed across educational and psychological traditions, but further empirical research is still needed to clarify how mathematically gifted students learn and develop their abilities. These recent studies suggest that mathematically gifted students should be examined not only in terms of cognitive characteristics, but also in relation to their learning processes and knowledge structures.

In addition to cognitive characteristics, recent studies have increasingly highlighted the importance of learning strategies, self-regulation, learning opportunities, and motivation among gifted students. Samnia Hammod and Paz-Baruch (2024) found that mathematically gifted junior high school students tended to use more self-regulated learning strategies when solving mathematical problems, especially when dealing with difficult tasks. Simensen and Olsen (2024) further showed that gifted students may benefit from rich mathematical tasks and adapted learning opportunities, particularly when they are encouraged to participate in meaningful mathematical discussions. Park et al. (2025) also indicated that growth math mindset, learning goals, intrinsic motivation, and mathematics outcomes are closely related in gifted students’ STEM learning contexts. These findings indicate that mathematics achievement among gifted students cannot be fully explained by mathematical content knowledge alone. Instead, it should also be examined in relation to students’ learning knowledge, learning strategies, self-regulation, motivation, and mathematics-specific learning processes.

Although previous studies have contributed to the understanding of mathematically gifted students’ cognitive characteristics, problem-solving abilities, creativity, self-regulated learning, and learning opportunities, relatively limited attention has been paid to their mathematical learning knowledge structures. In particular, there is still insufficient empirical evidence on how different types of learning-related knowledge are organized among mathematically gifted students and how these knowledge structures are associated with mathematics achievement. This gap is especially important in the Chinese junior high school context, where mathematics learning is strongly influenced by curriculum requirements, examination performance, classroom instruction, and students’ individual learning strategies. Therefore, there is a need to examine mathematically gifted students from a learner-centered knowledge-structure perspective.

The theoretical foundation of the present study is related to the development of Pedagogical Content Knowledge (PCK) and Mathematical Pedagogical Content Knowledge (MPCK). Shulman (1986) first proposed the concept of PCK to explain the special form of professional knowledge that teachers use to transform subject matter knowledge into forms that are understandable to students. Shulman (1987) further emphasized that PCK involves the integration of subject matter knowledge and pedagogical knowledge in relation to students’ interests, abilities, and learning difficulties. In mathematics education, Huang and Xu (2009) developed the concept of MPCK, which refers to the integration of Mathematical Knowledge (MK), Pedagogical Knowledge (PK), and Content Knowledge (CK) required for effective mathematics teaching. These frameworks have greatly contributed to understanding teachers’ professional knowledge in mathematics education.

However, the present study focuses not on teachers’ knowledge for teaching, but on students’ knowledge for learning mathematics. Building on the MPCK framework, Hao (2014) proposed the concept of Mathematics Learning Content Knowledge (MLCK), which shifted the focus from teachers’ knowledge structures to students’ knowledge structures. Hao’s MLCK framework includes Mathematical Knowledge (MK), Learning Knowledge (LK), and Content Knowledge (CK) related to mathematics learning. This shift is valuable because it recognizes that students also need a structured body of knowledge to support mathematics learning. Nevertheless, in the context of students’ mathematics learning, the distinction between LK and CK remains insufficiently clear. Some elements classified as CK, such as learning characteristics, learning strategies, and learning-related factors, may overlap with LK. This conceptual overlap may reduce the clarity and empirical operability of the framework when it is used to examine students’ mathematics learning.

To address this limitation, the present study refines the MLCK framework by introducing Mathematical Learning Knowledge (MLK) as a more focused and student-centered analytical dimension. In this study, Mathematical Knowledge (MK) refers to students’ knowledge of mathematical concepts, formulas, theorems, mathematical thinking methods, mathematical connections, and mathematical history and culture. Learning Knowledge (LK) refers to students’ general knowledge about learning, including learning methods, learning strategies, and understanding of learning processes that may apply across subjects. Mathematical Learning Knowledge (MLK), by contrast, refers specifically to mathematics-related learning knowledge. It includes students’ knowledge of how to select mathematical learning strategies, organize mathematical problem-solving processes, regulate learning when facing mathematical difficulties, and connect mathematical content with suitable learning methods. Therefore, MLK differs from MK because it is not limited to mathematical content itself, and it differs from LK because it focuses specifically on learning mathematics rather than learning in general.

The introduction of MLK provides a more precise framework for examining how mathematically gifted students learn mathematics. Mathematically gifted students often have relatively strong mathematical foundations, but their achievement may depend on how they understand learning, apply mathematics-specific strategies, regulate problem-solving processes, and connect mathematical knowledge with learning methods. From this perspective, mathematics achievement may be more closely related to students’ learning-related and mathematics-specific learning-related knowledge than to gender or mathematical knowledge alone. Therefore, examining MK, LK, and MLK together may provide a more comprehensive understanding of the mathematical learning knowledge structures of junior high school mathematically gifted students.

The present study is significant in three ways. First, it extends previous research on mathematically gifted students by moving beyond cognitive characteristics and mathematical achievement to examine students’ internal mathematical learning knowledge structures. Second, it refines the existing MLCK framework by replacing the less clearly distinguished CK dimension with MLK, which is more directly related to students’ mathematics-specific learning processes. Third, it provides empirical evidence from junior high school mathematically gifted students in Nanning, China, thereby contributing to the understanding of how MK, LK, and MLK are associated with mathematics achievement in a specific educational context.

Based on the above background, the purpose of this study is to investigate the mathematical learning knowledge structures of junior high school mathematically gifted students and to examine the relationship between these structures and mathematics achievement. Specifically, this study focuses on three dimensions: Mathematical Knowledge (MK), Learning Knowledge (LK), and Mathematical Learning Knowledge (MLK). It also examines whether gender differences exist in these dimensions and whether MK, LK, and MLK are associated with students’ mathematics achievement. Through this investigation, the study aims to provide theoretical and practical insights for understanding and supporting the mathematics learning of mathematically gifted students.

### MPCK (mathematical pedagogical content knowledge)

Huang and Xu (2009) have conducted extensive research on the MPCK (Mathematical Pedagogical Content Knowledge) required by teachers for mathematics instruction. They defined the structural model for MPCK as the integration of MK (Mathematical Knowledge), PK (Pedagogical Knowledge), and CK (Content Knowledge). They argued that mathematics teachers require more than just mathematical knowledge and also need the ability to effectively convey this knowledge and assist students in comprehending and applying it.

**FIGURE 1:**
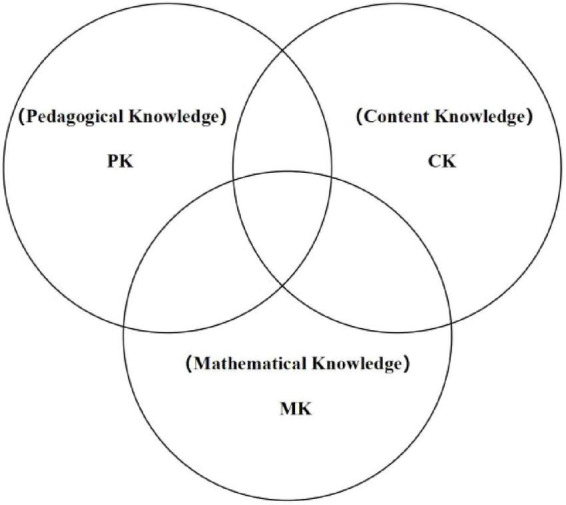
MPCK (mathematical pedagogical content knowledge) structures.

The MPCK structures diagram is shown in Figure 1, illustrating the comprehensive knowledge structures required by teachers for mathematics teaching.

### MLCK (mathematics learning content knowledge)

**FIGURE 2:**
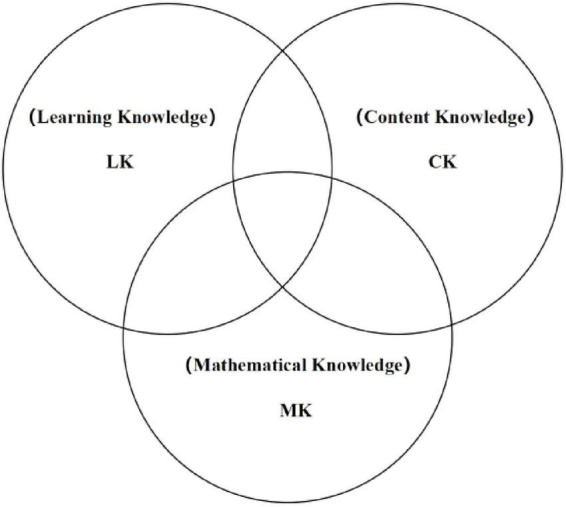
MLCK (mathematical learning content knowledge) structures.

MLCK (Mathematics Learning Content Knowledge) structures refers to the combined knowledge of MK, LK, and CK. Figure 2 depicts the MLCK structures diagram constructed by Hao (2014).

According to Hao (2014), MK refers to the foundational disciplines of mathematics and related knowledge of mathematical history and mathematical culture. LK refers to knowledge related to learning, including learning strategies and learning methods. CK refers to knowledge related to mathematics learning, which includes characteristics of mathematics learning and factors influencing mathematics learning.

Although Hao (2014) made an important contribution by shifting the focus from teachers’ knowledge structures to students’ mathematical learning, the distinction between CK and LK in the MLCK framework remains insufficiently clear in the context of students’ mathematics learning. In particular, many elements classified as CK, such as learning characteristics, strategies, and learning-related influences, overlap substantially with LK. This conceptual overlap weakens both the clarity and the empirical operability of the framework. Therefore, rather than treating CK as a fully separate dimension, the present study introduces MLK as a more focused analytical dimension. MLK refers to mathematics-specific learning-related knowledge, that is, the knowledge students draw on when organizing, regulating, and engaging in mathematics learning. In this sense, the present study does not reject the MLCK framework, but refines it for a more student-centered and empirically workable analysis of mathematical learning knowledge.

### MLK (mathematical learning knowledge)

Building upon the structures of MPCK by Huang and Xu (2009) and the structures of MLCK by Hao (2014), this study proposes the framework of students’ mathematical learning knowledge, referring to the knowledge required by students in learning mathematics. (1) MK (Mathematical Knowledge) encompasses students’ basic mathematical knowledge, which refers to the extent of students’ mastery of mathematical concepts, formulas, and theorems; mathematical thinking methods, referring to the mathematical thinking methods implied in mathematical problems; mathematical connections, referring to students’ understanding of the connections between mathematics and other subjects; and knowledge of mathematical history, referring to their understanding of mathematical history and mathematical culture. (2) LK (Learning Knowledge) refers to knowledge of learning methods and learning strategies. (3) MLK (Mathematical Learning Knowledge) refers to mathematics-specific learning-related knowledge, including mathematical learning strategies, methods, and other factors influencing mathematical learning, mainly including school, family, and society. In defining the constituent elements of MK, LK, and MLK, this study draws on the relevant definitions and constituent elements of MK, LK, and CK by Zhu (2014) and Hao (2014) in their respective research. In this study, LK refers to students’ general knowledge about learning, such as general learning methods, learning strategies, and understanding of learning processes. By contrast, MLK refers to mathematics-specific learning-related knowledge, such as how students select mathematical learning strategies, organize mathematical problem-solving processes, and regulate learning when dealing with mathematical concepts, formulas, theorems, and problems.

**FIGURE 3:**
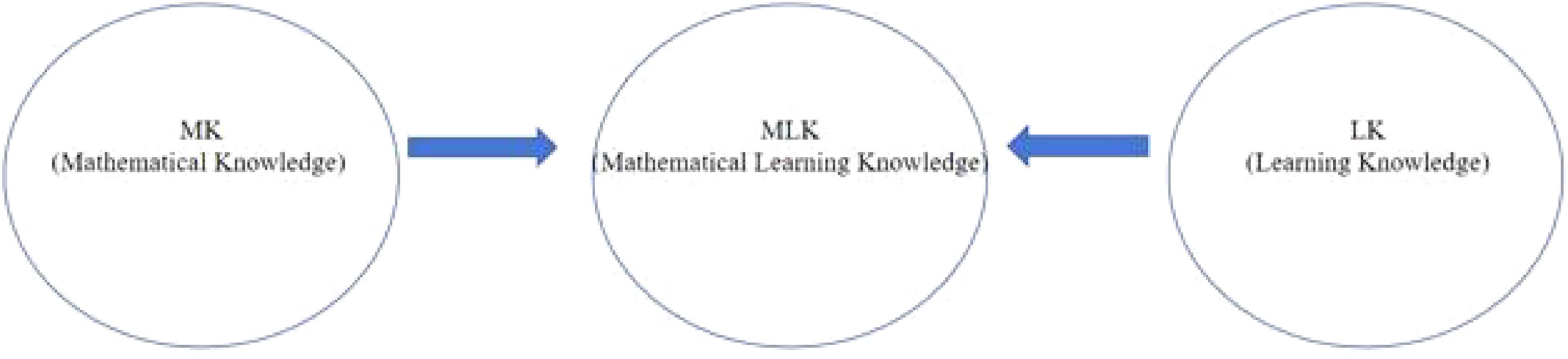
Conceptual framework of MK, LK, and MLK.

Based on the research by Huang and Xu (2009) on the MPCK structure, Hao (2014) replaced PK (Pedagogical Knowledge) with LK (Learning Knowledge) to construct the MLCK structure. This shift allows for the study of knowledge structures from the students’ perspective, providing ample theoretical foundation for this study. However, this study considers that the MLCK structure proposed by Hao (2014) has certain limitations, as separating CK from LK in the context of students’ mathematics learning seems somewhat inappropriate. Therefore, this study proposes a mathematical learning knowledge framework, as shown in Figure 3.

From the figure, it can be observed that the mathematical learning knowledge framework consists of LK (Learning Knowledge), MK (Mathematical Knowledge), and MLK (Mathematical Learning Knowledge). MLK refers to mathematics-specific learning-related knowledge and incorporates the unique characteristics of the mathematical discipline. Since the MLCK structure proposed by Hao (2014) has shifted the focus of knowledge structure research from teachers to students, the focus of the present study lies in the differences in the mathematical learning knowledge framework among mathematically gifted students and its relationship with mathematics achievement.

### Theoretical contribution of the MLK framework

The theoretical contribution of the present study lies in its reconceptualization of students’ mathematical learning knowledge from a learner-centered perspective. Existing PCK and MPCK frameworks mainly explain the knowledge teachers need in order to transform mathematical content for instruction. In contrast, the present study focuses on the knowledge students need when they learn, organize, regulate, and apply mathematics. Although Hao’s MLCK framework made an important contribution by shifting attention from teachers’ knowledge to students’ learning knowledge, the distinction between LK and CK remains conceptually overlapping in the context of student learning. The present study therefore refines this framework by introducing MLK as a mathematics-specific learning-related knowledge dimension.

MLK differs from MK because it does not refer simply to students’ mastery of mathematical concepts, formulas, theorems, mathematical thinking methods, or mathematical culture. It also differs from LK because it is not limited to general learning methods and strategies that may apply across school subjects. Instead, MLK refers to students’ knowledge of how to learn mathematics specifically, including how to select mathematical learning strategies, organize mathematical problem-solving processes, regulate learning when facing mathematical difficulties, and connect mathematical content with appropriate learning methods. Therefore, MLK provides a more focused construct for explaining how mathematically gifted students transform mathematical knowledge and general learning knowledge into mathematics-specific learning performance.

This refinement contributes to the literature by offering a more empirically workable framework for studying students’ mathematical learning knowledge structures. The regression results further support the relevance of this theoretical distinction, as MLK showed a significant positive association with mathematics achievement even after gender, MK, and LK were controlled.

## Research objectives

1. To describe the mathematical learning knowledge structures of junior high school mathematically gifted students in terms of MK, LK, and MLK.
2. To examine whether there are gender differences in MK, LK, MLK, and the overall mathematical learning knowledge structure.
3. To determine the extent to which gender, MK, LK, and MLK are associated with mathematics achievement among junior high school mathematically gifted students.

### Research hypotheses

This study divides the mathematical learning knowledge structures of junior high school mathematics gifted students into MK (Mathematical Knowledge), LK (Learning Knowledge), and MLK (Mathematical Learning Knowledge). Building upon this framework, two hypotheses are proposed:

*H1*: There are statistically significant gender differences in MK, LK, MLK, and the overall mathematical learning knowledge structure among junior high school mathematically gifted students.

*H2*: Gender, MK, LK, and MLK are significantly associated with mathematics achievement among junior high school mathematically gifted students.

### Research subjects and type

This study adopted a quantitative cross-sectional survey design. Specifically, it involved descriptive, comparative, and correlational components. The descriptive component was used to describe the mathematical learning knowledge structures of junior high school mathematically gifted students in terms of Mathematical Knowledge (MK), Learning Knowledge (LK), and Mathematical Learning Knowledge (MLK). The comparative component was used to examine whether gender differences existed in these dimensions. The correlational component was used to determine the relationship between the dimensions of mathematical learning knowledge structures and students’ mathematics achievement.

The target population of this study was junior high school mathematically gifted students in Nanning, China. The accessible population consisted of Grade 9 students from a key junior high school in Nanning. This school was selected because it has a strong academic atmosphere, a disciplined learning environment, and students with relatively strong foundations in mathematics learning. These characteristics made the school suitable for identifying and studying mathematically gifted students at the junior high school level.

In this study, mathematically gifted students were defined as students who showed strong interest in mathematics, good memory for mathematical concepts, formulas, and theorems, relatively high academic performance in mathematics, and strong abilities in problem solving, abstract reasoning, identifying mathematical models, and discovering mathematical relationships. The identification of mathematically gifted students was based on students’ mathematics academic records, mathematics examination results, class ranking records, and the professional judgment of mathematics teachers.

A criterion-based purposive sampling technique was used to select the participants. Nine Grade 9 classes were included in the study. With the assistance of the mathematics teachers from each class, students who consistently ranked among the top ten in mathematics were identified as mathematically gifted students. Therefore, ten students were selected from each of the nine classes, resulting in a total sample of 90 students. The final sample consisted of 45 female students and 45 male students.

The sample size was considered appropriate for the purpose and statistical analyses of the study. Since the study aimed to compare gender differences and examine the relationship between mathematical learning knowledge structures and mathematics achievement, a sample of 90 students was sufficient for conducting independent-samples *t*-tests and multiple linear regression analysis with gender, MK, LK, and MLK as predictors. The use of clear selection criteria and teacher confirmation also helped ensure that the participants were consistent with the definition of mathematically gifted students adopted in this study.

Overall, the selected participants represented Grade 9 mathematically gifted students from the participating junior high school. Although the sample was limited to one school, the selection procedure was transparent and consistent with the purpose of the study, which was to examine the mathematical learning knowledge structures of junior high school mathematically gifted students and their relationship with mathematics achievement.

### Ethical considerations

Ethical considerations were carefully addressed in this study. Before data collection, this study obtained ethical approval from the Scientific Research Ethics Committee of Nanning Normal University (Approval No. LLSH2024-024, approved on May 13, 2024). The research procedures complied with ethical principles and relevant regulations. Permission was also obtained from the participating school before the questionnaire survey and mathematics test were conducted.

Since the participants were junior high school students, the purpose and procedures of the study were explained to the students, their parents or guardians, and the relevant school personnel before data collection. Participation in the study was voluntary. Informed consent was obtained from the students’ parents or guardians, and assent was obtained from the students. The students were informed that the collected data would be used only for academic research purposes and that they could withdraw from the study at any time without any negative consequences.

To protect participants’ privacy, no personally identifiable information was reported in this study. All data were processed anonymously and kept confidential. The results were presented only in aggregate form, and no individual student could be identified from the reported findings.

## Research materials and methods

### Research design

This study adopted a quantitative cross-sectional survey design. It was descriptive, comparative, and correlational in nature. The descriptive component was used to examine the mathematical learning knowledge structures of junior high school mathematically gifted students in terms of Mathematical Knowledge (MK), Learning Knowledge (LK), and Mathematical Learning Knowledge (MLK). The comparative component was used to examine whether there were gender differences in MK, LK, MLK, and the overall mathematical learning knowledge structure. The correlational component was used to investigate the relationship between the dimensions of mathematical learning knowledge structures and students’ mathematics achievement.

### Participants and sampling

The target population of this study was junior high school mathematically gifted students in Nanning, China. The accessible population consisted of Grade 9 students from a key junior high school in Nanning. This school was selected because it has a strong academic atmosphere, a disciplined learning environment, and students with relatively strong foundations in mathematics learning.

A criterion-based purposive sampling technique was used to select the participants. Nine Grade 9 classes were included in the study. With the assistance of the mathematics teachers from each class, students who consistently ranked among the top ten in mathematics were identified as mathematically gifted students. Ten students were selected from each of the nine classes, resulting in a total sample of 90 students. The final sample consisted of 45 female students and 45 male students.

In this study, mathematically gifted students were defined as students who showed strong interest in mathematics, relatively high mathematics achievement, good memory for mathematical concepts, formulas, and theorems, and strong abilities in problem solving, abstract reasoning, identifying mathematical models, and discovering mathematical relationships. The identification of the participants was based on their mathematics academic performance, class ranking records, and the professional judgment of their mathematics teachers.

The sample size was considered appropriate for the purpose of the study and the statistical analyses conducted. Since the study aimed to compare gender differences and examine the relationship between mathematical learning knowledge structures and mathematics achievement, a sample of 90 students was sufficient for conducting independent-samples *t*-tests and multiple linear regression analysis with gender, MK, LK, and MLK as predictors.

### Research instruments

Two instruments were used in this study: the Mathematical Learning Knowledge Questionnaire and the Mathematics Achievement Test. The questionnaire was used to collect data on students’ mathematical learning knowledge structures, while the mathematics test was used to assess students’ mathematics achievement.

### Mathematical learning knowledge questionnaire

The Mathematical Learning Knowledge Questionnaire was adapted from Hao’s (2014) study on mathematical learning knowledge structures. The original framework was modified to suit the learning characteristics and educational context of junior high school mathematically gifted students. In particular, the present study focused on three dimensions: Mathematical Knowledge (MK), Learning Knowledge (LK), and Mathematical Learning Knowledge (MLK).

This modification was made because the distinction between Learning Knowledge and Content Knowledge in the original MLCK framework may overlap in the context of students’ mathematics learning. Therefore, Mathematical Learning Knowledge was introduced as a more focused dimension to represent students’ mathematics-specific learning-related knowledge.

The questionnaire consisted of 60 items. The structure of the questionnaire is shown in Table 1. Each of the three dimensions contained 20 items. The MK dimension measured students’ knowledge of mathematical concepts, formulas, theorems, mathematical thinking methods, mathematical connections, and mathematical history and culture. The LK dimension measured students’ general knowledge of learning, including understanding of learning, learning methods, and learning strategies. The MLK dimension measured students’ mathematics-specific learning knowledge, including mathematical learning methods, mathematical learning strategies, and factors influencing mathematics learning.

**TABLE 1:** Structure of the mathematical learning knowledge questionnaire.

Dimension	Sub-dimensions	Number of Items	Scoring
MK	Mathematical history and culture; mathematical connections; mathematical thinking methods; mathematical concepts, formulas, and theorems	20	1 = correct; 0 = incorrect
LK	Understanding of learning; learning methods; learning strategies	20	1 = correct; 0 = incorrect
MLK	Mathematical learning methods; mathematical learning strategies; influential factors on mathematical learning	20	1 = correct; 0 = incorrect
Total	–	60	Higher scores indicate stronger mastery

MK, Mathematical Knowledge; LK, Learning Knowledge; MLK, Mathematical Learning Knowledge.

All questionnaire items were scored dichotomously. Each correct answer was given 1 point, while each incorrect answer was given 0 points. A higher score indicated a stronger level of mastery of the corresponding dimension of mathematical learning knowledge structure.

### Mathematics achievement test

The Mathematics Achievement Test was developed for the present study to assess students’ mathematics achievement. The test questions covered junior high school mathematics topics, including triangles and polynomials. The test was designed based on Bloom’s (1956) cognitive classification and included four dimensions: computation, comprehension, application, and analysis.

The test consisted of 60 questions, with a total score of 100 points. The computation dimension included questions assessing students’ ability in practical calculations. The comprehension dimension included questions assessing students’ ability to transform problem forms. The application dimension included questions assessing students’ ability to make comparisons and solve routine problems. The analysis dimension included questions assessing students’ ability to discern mathematical relationships and construct proofs.

The computation and comprehension dimensions each contained 10 questions, with each question worth 1 point. The application and analysis dimensions each contained 20 questions, with each question worth 2 points. Therefore, the total score of the Mathematics Achievement Test was 100 points. The structure of the Mathematics Achievement Test is shown in Table 2.

**TABLE 2:** Structure of the mathematics achievement test.

Dimension	Sub-dimensions	Number of questions	Score
Computation	Ability in practical calculations	10	10
Comprehension	Ability to transform problem forms	10	10
Application	Ability to make comparisons; ability to solve routine problems	20	40
Analysis	Ability to discern relationships; ability to construct proofs	20	40
Total	–	60	100

The test consisted of 60 questions with a total score of 100 points.

### Validity of the instruments

Content validity was used to examine whether the instruments adequately represented the constructs measured in this study. Content validity concerns the extent to which the items of an instrument adequately represent the construct that the instrument is intended to measure (Yaghmaei, 2003). Therefore, expert review was used in this study to examine whether the questionnaire and mathematics test appropriately represented the intended dimensions and were suitable for junior high school mathematically gifted students. To establish the content validity of the Mathematical Learning Knowledge Questionnaire and the Mathematics Achievement Test, three experts in mathematics education and educational research from Nanning Normal University were invited to review the instruments.

The experts evaluated the relevance, representativeness, clarity, and suitability of the items. Specifically, they examined whether the questionnaire items appropriately reflected the dimensions of MK, LK, and MLK, and whether the mathematics test items adequately represented the dimensions of computation, comprehension, application, and analysis. They also assessed whether the wording, item arrangement, and difficulty level were suitable for junior high school mathematically gifted students.

Based on the experts’ comments and suggestions, revisions were made to improve the clarity, wording, organization, and suitability of the instruments. Ambiguous expressions were revised, item arrangement was adjusted, and items that required clarification were modified. After these revisions, the instruments were considered to have acceptable content validity for use in the present study.

### Reliability of the instruments

The reliability of the instruments was examined using internal consistency reliability. Internal consistency reliability was examined using Cronbach’s alpha, which is commonly used to assess the extent to which items within an instrument consistently measure the same construct (Tang et al., 2014). According to George and Mallery (2019), Cronbach’s alpha values above.70 are generally considered acceptable, while higher values indicate stronger internal consistency. Cronbach’s alpha coefficients were calculated using SPSS version 26. The reliability analysis showed that the instruments had acceptable to high internal consistency. The Cronbach’s alpha coefficients are shown in Table 3.

**TABLE 3:** Cronbach’s alpha coefficients for internal consistency of the questionnaire and mathematics test.

Instrument	Number of items	Cronbach’s alpha
Mathematical learning knowledge questionnaire	60	0.948
MK subscale	20	0.866
LK subscale	20	0.862
MLK subscale	20	0.886
Mathematics achievement test	60	0.827

MK, Mathematical Knowledge; LK, Learning Knowledge; MLK, Mathematical Learning Knowledge.

The Cronbach’s alpha coefficient for the overall Mathematical Learning Knowledge Questionnaire was 0.948. For the three subscales, the Cronbach’s alpha coefficients were 0.866 for MK, 0.862 for LK, and 0.886 for MLK. The Cronbach’s alpha coefficient for the Mathematics Achievement Test was 0.827. These values indicate that the questionnaire and mathematics test had acceptable reliability for the purposes of the study.

### Data collection procedure

Data collection was conducted in two stages. The first stage was the survey stage. During this stage, the Mathematical Learning Knowledge Questionnaire was administered to the selected junior high school mathematically gifted students. The purpose of this stage was to collect data on students’ MK, LK, and MLK.

The second stage was the test stage. During this stage, the Mathematics Achievement Test was administered to the same group of students. The purpose of this stage was to assess students’ mathematics achievement. Before data collection, the purpose and procedures of the study were explained to the students and relevant school personnel. The questionnaire and mathematics test were administered under standardized instructions. All responses and test scores were collected and prepared for statistical analysis.

### Data analysis

The collected data were analyzed using SPSS version 26. Descriptive statistics, including means and standard deviations, were used to describe students’ MK, LK, MLK, overall mathematical learning knowledge structure, and mathematics achievement.

Independent-samples *t*-tests were conducted to examine whether there were significant gender differences in MK, LK, MLK, and the overall mathematical learning knowledge structure. Multiple linear regression analysis was conducted to examine whether gender, MK, LK, and MLK were associated with mathematics achievement. In the regression model, mathematics achievement was treated as the dependent variable, while gender, MK, LK, and MLK were treated as independent variables. The level of statistical significance was set at *p* < 0.05.

## Results and analysis

This section presents the statistical results according to the research hypotheses. Each hypothesis is stated before the corresponding analysis in order to improve the organization and clarity of the findings. The interpretation focuses on the meaning of the statistical results in relation to the research objectives rather than merely repeating the values shown in the tables.

### Hypothesis 1: gender differences in mathematical learning knowledge structures

H1 stated that there are statistically significant gender differences in Mathematical Knowledge (MK), Learning Knowledge (LK), Mathematical Learning Knowledge (MLK), and the overall mathematical learning knowledge structure among junior high school mathematically gifted students. To test this hypothesis, independent-samples *t*-tests were conducted.

As shown in Table 4, female students obtained slightly higher mean scores than male students across MK, LK, MLK, and the overall mathematical learning knowledge structure. However, none of these differences reached statistical significance, as all *p*-values were greater than 0.05. These results indicate that gender did not significantly differentiate the mathematical learning knowledge structures of the mathematically gifted students in this sample. Therefore, H1 was not supported.

**TABLE 4:** Independent-samples *t*-test results for gender differences in MK, LK, MLK, and Overall MLKS.

Variable	Female (*n* = 45) M (SD)	Male (*n* = 45) M (SD)	*t*	df	*P*	Decision
MK	16.4222 (3.99140)	15.4667 (4.49039)	1.067	88	0.289	Not significant
LK	16.2444 (3.73044)	15.4889 (4.71758)	.843	88	0.402	Not significant
MLK	17.0000 (3.61814)	15.7333 (4.91010)	1.393	80.903	0.167	Not significant
Overall MLKS	49.6667 (10.07698)	46.6889 (13.32943)	1.195	81.911	0.235	Not significant

MK, Mathematical Knowledge; LK, Learning Knowledge; MLK, Mathematical Learning Knowledge; MLKS, Mathematical Learning Knowledge Structure. For MK and LK, *p* values were based on equal variances assumed. For MLK and Overall MLKS, *p* values were based on equal variances not assumed.

### Hypothesis 2: relationship between mathematical learning knowledge structures and mathematics achievement

H2 stated that gender, MK, LK, and MLK are significantly associated with mathematics achievement among junior high school mathematically gifted students. To test this hypothesis, a multiple linear regression analysis was conducted with mathematics achievement as the dependent variable and gender, MK, LK, and MLK as independent variables.

Table 5 shows that the overall regression model was statistically significant, *F*(4, 85) = 33.598, *p* < 0.001. The model explained 61.3% of the variance in mathematics achievement, with an adjusted R^2^ of 0.594. This indicates that gender, MK, LK, and MLK, when considered together, had substantial explanatory power for mathematics achievement among the mathematically gifted students.

**TABLE 5:** Model summary for multiple linear regression predicting mathematics achievement.

*R*	*R* ^2^	Adjusted R^2^	SE of estimate	*F*	df	*p*
0.783	0.613	0.594	5.762	33.598	4.85	< 0.001

Dependent variable = mathematics achievement test score.

As shown in Table 6, LK was a significant positive predictor of mathematics achievement (*B* = 0.768, β = 0.360, *p* = 0.003). MLK was also a significant positive predictor (*B* = 0.679, β = 0.325, *p* = 0.017). These findings indicate that students with higher levels of general learning knowledge and mathematics-specific learning knowledge tended to obtain higher mathematics achievement scores after gender and the other knowledge-structure dimensions were controlled.

**TABLE 6:** Regression coefficients for predictors of mathematics achievement.

Predictor	*B*	SE	β	*t*	*p*	95% CI for B	Tolerance	VIF
Constant	62.543	2.722	–	22.979	< 0.001	[57.132, 67.955]	–	–
Gender	−0.609	1.230	−0.034	−0.495	0.622	[−3.054, 1.836]	0.976	1.025
MK	0.321	0.244	0.151	1.315	0.192	[−0.164, 0.806]	0.346	2.886
LK	0.768	0.252	0.360	3.043	0.003	[0.266, 1.269]	0.325	3.074
MLK	0.679	0.278	0.325	2.441	0.017	[0.126, 1.231]	0.257	3.894

MK, Mathematical Knowledge; LK, Learning Knowledge; MLK, Mathematical Learning Knowledge. Gender was coded as 0, female and 1, male. CI, confidence interval.

By contrast, gender was not a significant predictor of mathematics achievement (*p* = 0.622). MK was also not a significant unique predictor in the full regression model (*p* = 0.192), although it was positively related to achievement at the descriptive and bivariate levels in the original analysis. This suggests that, when LK and MLK were included in the model, the unique contribution of mathematical knowledge alone was reduced. The tolerance values ranged from 0.257 to 0.976 and the VIF values ranged from 1.025 to 3.894, indicating that severe multicollinearity was not present.

Overall, the regression results provided partial support for H2. The overall model was significant, and LK and MLK were significantly and positively associated with mathematics achievement. However, gender and MK were not significant unique predictors after the other variables were controlled. Therefore, the findings suggest that mathematics achievement among junior high school mathematically gifted students was more closely associated with learning-related and mathematics-specific learning-related knowledge than with gender or mathematical knowledge alone.

## Discussion

The purpose of this study was to investigate the mathematical learning knowledge structures of junior high school mathematically gifted students and to examine the relationship between these structures and mathematics achievement. Specifically, the study focused on three dimensions: Mathematical Knowledge (MK), Learning Knowledge (LK), and Mathematical Learning Knowledge (MLK). The findings provide useful insights into how mathematically gifted students learn mathematics and how different dimensions of their mathematical learning knowledge structures are associated with mathematics achievement.

### Gender differences in mathematical learning knowledge structures

The first hypothesis proposed that there would be gender-based differences in the mathematical learning knowledge structures of junior high school mathematically gifted students. However, the results of the independent-samples t-tests showed that there were no statistically significant gender differences in MK, LK, MLK, or the overall mathematical learning knowledge structure. Although female students obtained slightly higher mean scores than male students across the measured dimensions, these differences were not statistically significant. Therefore, the first hypothesis was not supported.

This finding suggests that gender may not be a determining factor in differentiating the mathematical learning knowledge structures of junior high school mathematically gifted students. One possible explanation is that the participants in this study were selected from the top mathematics performers in each class. Since both male and female students in the sample had relatively strong mathematics achievement and learning foundations, gender differences may have been reduced within this high-performing group. In other words, when students are already identified as mathematically gifted, their mathematical learning knowledge structures may be more strongly shaped by their learning experiences, learning strategies, and mathematics-related engagement than by gender itself.

This finding extends previous research on mathematically gifted students by suggesting that, within a high-performing gifted group, gender may not be the main factor differentiating students’ mathematical learning knowledge structures. Previous studies have emphasized that mathematically gifted students are often characterized by distinctive mathematical reasoning, flexible thinking, rapid problem solving, and advanced mathematical engagement rather than by gender-based characteristics (Koshy et al., 2009; Leikin, 2010; Singer et al., 2016). Recent studies have also highlighted the multidimensional nature of mathematical giftedness and the importance of learning processes and learning opportunities (Sipahi and Bahar, 2024; Özdemir et al., 2024). Therefore, the absence of significant gender differences in the present study is consistent with the view that support for mathematically gifted students should focus more on their learning characteristics, strategy use, and mathematics-specific learning needs than on gender assumptions.

This result also has important educational implications. It suggests that teachers should avoid making gender-based assumptions about students’ mathematical learning ability, learning strategies, or mathematical potential. Instead, both male and female mathematically gifted students should be provided with equal opportunities to develop mathematical thinking, learning strategies, and mathematics-specific learning knowledge. From the perspective of equity in mathematics education, the absence of significant gender differences indicates that mathematics learning support for gifted students should be based on students’ actual learning needs rather than on gender stereotypes.

### Relationship between mathematical learning knowledge structures and mathematics achievement

The second hypothesis proposed that the dimensions of mathematical learning knowledge structures would be associated with mathematics achievement. The multiple regression results provided partial support for this hypothesis. The overall regression model was statistically significant and explained a substantial proportion of the variance in mathematics achievement. This indicates that gender, MK, LK, and MLK, when considered together, were meaningfully associated with students’ mathematics test scores.

More specifically, LK and MLK were significant positive predictors of mathematics achievement. This means that students with stronger learning knowledge and mathematics-specific learning knowledge tended to achieve higher mathematics test scores. This finding supports the view that mathematics achievement among mathematically gifted students is not determined only by the amount of mathematical content knowledge they possess. Rather, achievement is also closely related to how students understand learning, organize their learning processes, select learning strategies, and apply mathematics-specific methods when solving mathematical problems.

The significant role of LK indicates that general learning knowledge remains important even among mathematically gifted students. Students who understand how to learn, how to use learning methods, and how to apply learning strategies may be better able to manage their mathematics learning. Such students may be more capable of previewing, reviewing, summarizing, classifying knowledge points, monitoring their understanding, and adjusting their learning strategies when they encounter difficulties. These general learning abilities can support students in developing more effective and independent learning habits.

The significant role of MLK is particularly important because it reflects the mathematics-specific nature of learning. MLK is not simply general learning knowledge, nor is it only mathematical content knowledge. Instead, it refers to students’ knowledge of how to learn mathematics specifically. This includes knowing how to choose appropriate mathematical learning strategies, organize mathematical problem-solving processes, connect mathematical concepts with formulas and theorems, and regulate learning when dealing with challenging mathematical tasks. The finding that MLK significantly predicted mathematics achievement suggests that mathematically gifted students benefit from being able to connect mathematical content with suitable learning processes.

This finding is consistent with recent studies emphasizing the importance of learning processes among mathematically gifted students. Samnia Hammod and Paz-Baruch (2024) found that mathematically gifted junior high school students used self-regulated learning strategies when solving mathematical problems, particularly when dealing with difficult tasks. Simensen and Olsen (2024) also showed that rich mathematical tasks and meaningful mathematical discussion can help gifted students actualize their mathematical potential. In addition, Park et al. (2025) reported that growth math mindset, learning goals, intrinsic motivation, and mathematics outcomes are closely related in gifted students’ STEM learning contexts. These studies support the present finding that mathematics achievement among mathematically gifted students is closely related to learning strategies, self-regulation, and mathematics-specific learning processes. For example, mathematically gifted students may not rely only on quick calculation or strong memory; they also need to monitor their thinking, select appropriate strategies, and reflect on their problem-solving processes. Therefore, mathematics achievement should be understood as the result of both mathematical knowledge and the ability to manage mathematics learning effectively.

### The non-significant unique role of mathematical knowledge

An important finding of this study is that MK was positively correlated with mathematics achievement at the bivariate level but was not a significant unique predictor in the full regression model after gender, LK, and MLK were controlled. This result does not mean that mathematical knowledge is unimportant. Rather, it suggests that the unique contribution of MK may overlap with the contributions of LK and MLK.

This finding both aligns with and extends previous research. Prior studies have shown that mathematically gifted students often possess strong mathematical reasoning, rapid generalization ability, and advanced problem-solving characteristics (Krutetskii, 1976; Koshy et al., 2009; Leikin and Lev, 2012; Singer et al., 2016). However, the present study further shows that mathematical knowledge alone may not be sufficient to explain achievement differences within a group of mathematically gifted students. This differs from a narrow interpretation that high mathematical knowledge directly leads to high mathematics achievement. Instead, the results suggest that students’ ability to organize, regulate, and apply mathematical knowledge through general learning knowledge and mathematics-specific learning knowledge may play a more important role in explaining achievement differences among mathematically gifted students.

One possible explanation is that the participants in this study were mathematically gifted students who generally had relatively strong mathematical foundations. Since these students were selected based on their high mathematics performance, there may have been less variation in their basic mathematical knowledge compared with a general student population. As a result, MK alone may not have been sufficient to explain differences in mathematics achievement within this group. For mathematically gifted students, differences in achievement may depend more on how they use, organize, and apply their mathematical knowledge during learning and problem solving.

Another possible explanation is that MK may influence mathematics achievement indirectly through LK and MLK. Students who possess mathematical knowledge may still need effective learning methods and mathematics-specific learning strategies to transform that knowledge into strong performance. For example, knowing mathematical formulas and theorems does not automatically lead to high achievement if students cannot select appropriate problem-solving strategies, identify relationships among concepts, or regulate their learning when facing difficult tasks. Therefore, the non-significant unique effect of MK in the regression model highlights the importance of learning-related and mathematics-specific learning-related knowledge in explaining achievement among mathematically gifted students.

This finding also supports the theoretical value of distinguishing MK, LK, and MLK. If mathematics achievement were explained only by mathematical content knowledge, then MK would have been expected to remain a strong unique predictor. However, the results showed that LK and MLK were more important unique predictors in the full model. This suggests that the proposed mathematical learning knowledge framework provides a useful way to understand the learning characteristics of mathematically gifted students.

### Theoretical implications

The findings of this study contribute to the literature on mathematically gifted students and mathematical learning knowledge structures. Previous research on mathematical giftedness has often focused on cognitive characteristics, creativity, reasoning ability, or problem-solving performance. While these aspects are important, the present study shows that students’ internal learning knowledge structures also deserve attention. In particular, the significant effects of LK and MLK indicate that mathematical giftedness should be examined not only from the perspective of ability, but also from the perspective of learning knowledge and learning processes.

The results also support the refinement of the MLCK framework. Hao’s framework shifted the focus from teachers’ knowledge structures to students’ learning knowledge structures, which provided an important foundation for this study. However, the present study further refined this framework by introducing MLK as a mathematics-specific learning-related dimension. The regression results provide empirical support for this refinement because MLK was significantly associated with mathematics achievement. This indicates that mathematics-specific learning knowledge is a meaningful construct for understanding how mathematically gifted students learn and perform in mathematics.

This theoretical implication is also related to the development of knowledge-structure research in mathematics education. Shulman’s (1986, 1987) PCK framework and Huang and Xu’s (2009) MPCK framework mainly explained the knowledge needed by teachers for effective instruction. Hao’s (2014) MLCK framework made an important shift by focusing on students’ knowledge for mathematics learning. The present study extends this line of research by refining the CK dimension into MLK, a more mathematics-specific and learner-centered construct. The finding that MLK significantly predicted mathematics achievement provides empirical support for this refinement and suggests that students’ mathematics-specific learning knowledge is a meaningful dimension for understanding mathematically gifted students’ achievement.

### Practical implications

The findings have practical implications for mathematics teaching and gifted education. First, teachers should not focus only on the transmission of mathematical concepts, formulas, and theorems. Although mathematical knowledge remains important, teachers should also help students develop effective learning methods, learning strategies, and self-regulated learning habits. This is especially important for mathematically gifted students, who may already have strong mathematical foundations but still need guidance in organizing and applying their knowledge effectively.

Second, teachers should provide opportunities for students to reflect on their mathematical learning processes. For example, students can be encouraged to explain how they solve problems, compare different solution strategies, summarize common problem types, and reflect on errors. These activities can help students develop stronger MLK by connecting mathematical content with learning strategies and problem-solving methods.

Third, schools and curriculum planners should pay more attention to the development of mathematics-specific learning knowledge in mathematics instruction. Enrichment activities for mathematically gifted students should not only include difficult mathematical problems, but also tasks that require students to plan, monitor, evaluate, and explain their mathematical thinking. Such activities can support the development of both LK and MLK and may contribute to higher mathematics achievement.

These practical implications are consistent with recent studies emphasizing that mathematically gifted students benefit from learning environments that support strategy use, self-regulation, and meaningful mathematical engagement. Samnia Hammod and Paz-Baruch (2024) showed that mathematically gifted students use self-regulated learning strategies during mathematical problem solving, while Simensen and Olsen (2024) emphasized the importance of rich mathematical tasks and mathematical discussion. Park et al. (2025) further indicated that motivation-related factors and mathematics outcomes are connected in gifted students’ STEM learning contexts. Therefore, classroom instruction and school-based support for mathematically gifted students should not only provide challenging mathematical content, but also intentionally cultivate students’ learning strategies, mathematical reflection, and mathematics-specific learning processes.

Overall, the findings suggest that supporting mathematically gifted students requires a balanced focus on mathematical knowledge, general learning knowledge, and mathematics-specific learning knowledge. Mathematics achievement among gifted students is not simply the result of possessing more mathematical knowledge. It is also closely related to how students learn, regulate, and apply mathematics. Therefore, the development of LK and MLK should be regarded as an important part of mathematics gifted education.

## Conclusion

This study examined the mathematical learning knowledge structures of junior high school mathematically gifted students in Nanning, China, with a focus on three dimensions: Mathematical Knowledge (MK), Learning Knowledge (LK), and Mathematical Learning Knowledge (MLK). It also investigated gender differences in these knowledge structures and their relationship with mathematics achievement.

The findings showed that there were no statistically significant gender differences in MK, LK, MLK, or the overall mathematical learning knowledge structure. This indicates that gender was not a significant factor in differentiating the mathematical learning knowledge structures of the mathematically gifted students in this study.

The regression results further showed that the overall model significantly predicted mathematics achievement. Among the predictors, LK and MLK were significant positive predictors of mathematics achievement, whereas gender and MK were not significant unique predictors after controlling for the other variables. These findings suggest that mathematics achievement among junior high school mathematically gifted students is more closely associated with learning-related knowledge and mathematics-specific learning knowledge than with gender or mathematical knowledge alone.

Overall, the study highlights the importance of developing students’ general learning knowledge and mathematics-specific learning knowledge in mathematics gifted education. Teachers should therefore not only strengthen students’ mathematical content knowledge, but also guide them to develop effective learning strategies, regulate their mathematical learning processes, and connect mathematical knowledge with appropriate learning methods.

### Recommendations

Based on the findings of this study, several recommendations are proposed for students, mathematics teachers, school administrators, curriculum planners, and educational policymakers. Since the results showed that Learning Knowledge (LK) and Mathematical Learning Knowledge (MLK) were significant positive predictors of mathematics achievement, the recommendations mainly focus on strengthening students’ learning strategies, mathematics-specific learning methods, and self-regulated learning processes. In addition, because no significant gender differences were found in mathematical learning knowledge structures, the recommendations also emphasize equal learning support for both male and female mathematically gifted students.

### Recommendations for students

Students should not rely only on memorizing mathematical concepts, formulas, and theorems. Although Mathematical Knowledge (MK) remains important, the findings of this study suggest that mathematics achievement is more closely associated with how students understand, organize, and apply learning strategies. Therefore, students should develop effective general learning habits, such as previewing lessons, reviewing key points, summarizing knowledge, classifying problem types, and reflecting on mistakes after solving problems.

Mathematically gifted students should also pay particular attention to Mathematical Learning Knowledge (MLK). This means that they should learn how to select suitable strategies for different types of mathematical problems, connect mathematical concepts with formulas and theorems, compare different solution methods, and monitor their thinking during problem solving. By consciously developing mathematics-specific learning strategies, students can improve their ability to transform mathematical knowledge into stronger mathematics achievement.

### Recommendations for mathematics teachers

Mathematics teachers should not focus only on the transmission of mathematical content knowledge. Since LK and MLK were found to be significant predictors of mathematics achievement, teachers should guide students to develop effective learning methods and mathematics-specific learning strategies. For example, teachers can explicitly teach students how to analyze problem conditions, identify mathematical relationships, select suitable solution strategies, summarize problem-solving patterns, and reflect on errors.

Teachers should also create opportunities for students to explain their mathematical thinking. Classroom activities such as solution comparison, group discussion, error analysis, learning reflection, and problem-solving reports can help students connect mathematical knowledge with learning strategies. These activities are particularly useful for developing students’ MLK.

In addition, since the study found no significant gender differences in MK, LK, MLK, or the overall mathematical learning knowledge structure, teachers should avoid making gender-based assumptions about students’ mathematical ability or learning potential. Both male and female students should be provided with equal opportunities to participate in mathematical discussion, problem-solving activities, enrichment tasks, and mathematics competitions.

### Recommendations for school administrators

School administrators should provide institutional support for the development of students’ learning knowledge and mathematical learning knowledge. Schools may establish mathematics learning groups, mathematics interest clubs, peer-learning communities, or enrichment programs for mathematically gifted students. These activities should not only provide more difficult mathematical problems, but also guide students to discuss learning methods, compare problem-solving strategies, and reflect on mathematical thinking.

Schools should also support teachers in designing activities that promote self-regulated learning and mathematics-specific learning strategies. Professional development workshops can be organized to help mathematics teachers understand how to cultivate students’ LK and MLK in daily classroom instruction. In addition, schools may encourage teachers to collect and share effective teaching cases related to mathematical learning strategies and gifted students’ learning development.

### Recommendations for curriculum planners

Curriculum planners should consider integrating learning strategies and mathematics-specific learning processes into mathematics curriculum design. The curriculum should not only emphasize mathematical concepts, formulas, theorems, and procedural skills, but also include activities that help students plan, monitor, and evaluate their mathematical learning.

For mathematically gifted students, curriculum enrichment should include rich mathematical tasks, open-ended problems, mathematical discussion, problem-solving reflection, and strategy comparison. These components can help students develop both general learning knowledge and mathematics-specific learning knowledge. Curriculum materials may also include guiding questions that encourage students to explain why a strategy is appropriate, how different mathematical ideas are connected, and how problem-solving processes can be improved.

### Recommendations for educational policymakers

Educational policymakers should give greater attention to the development of mathematically gifted students beyond test performance alone. The findings of this study suggest that mathematics achievement is closely related to students’ learning-related and mathematics-specific learning-related knowledge. Therefore, gifted education policies should support not only the identification of high-achieving students, but also the development of their learning strategies, self-regulation, and mathematics-specific learning competence.

Policy support may include teacher training programs on gifted mathematics education, differentiated curriculum resources, enrichment programs, and assessment systems that value mathematical thinking and learning processes. Policymakers should also encourage schools to provide equal support for both male and female mathematically gifted students, as the present study did not find significant gender differences in their mathematical learning knowledge structures.

Overall, the findings suggest that effective support for junior high school mathematically gifted students should move beyond a narrow focus on mathematical knowledge itself. Students’ learning knowledge and mathematical learning knowledge should be intentionally developed through classroom instruction, school-based support, curriculum design, and educational policy. Such efforts may help mathematically gifted students transform their mathematical potential into sustained mathematics achievement.

### Limitations and future research

Although this study provides useful insights into the mathematical learning knowledge structures of junior high school mathematically gifted students, several limitations should be acknowledged.

First, the participants were selected from only one key junior high school in Nanning, China. Although the school was suitable for identifying mathematically gifted students because of its strong academic atmosphere and students’ relatively strong mathematics foundations, the findings may not be fully generalizable to mathematically gifted students from other schools, regions, or educational contexts. Future studies should include larger and more diverse samples from different schools, cities, and provinces to improve the generalizability of the findings.

Second, the sample size was limited to 90 Grade 9 students. Although this sample size was sufficient for the statistical analyses conducted in the present study, a larger sample would provide stronger statistical power and allow for more advanced analyses. Future research may include a larger number of mathematically gifted students across different grade levels to examine whether mathematical learning knowledge structures differ by age, grade, or stage of mathematical development.

Third, the identification of mathematically gifted students was mainly based on students’ mathematics performance, class ranking records, and the professional judgment of mathematics teachers. Although these criteria were appropriate for the purpose of this study, they may not fully capture the multidimensional nature of mathematical giftedness. Future studies should consider using more comprehensive identification procedures, such as standardized mathematics ability tests, mathematical creativity assessments, problem-solving tasks, teacher nominations, and student learning portfolios.

Fourth, this study adopted a quantitative cross-sectional survey design. Therefore, the results can show associations among gender, MK, LK, MLK, and mathematics achievement, but they cannot establish causal relationships. Future studies may adopt longitudinal designs to examine how students’ mathematical learning knowledge structures develop over time. Experimental or intervention studies could also be conducted to investigate whether targeted training in learning strategies and mathematics-specific learning knowledge can improve students’ mathematics achievement.

Fifth, the Mathematics Achievement Test used in this study covered selected junior high school mathematics topics, including triangles and polynomials. Therefore, the test may not represent all areas of junior high school mathematics achievement. Future research should use broader achievement measures covering more mathematical domains, such as algebra, geometry, functions, statistics, and problem solving, in order to provide a more comprehensive assessment of students’ mathematics achievement.

Sixth, the present study mainly relied on questionnaire and test data. Although these instruments provided useful quantitative evidence, they may not fully explain how mathematically gifted students actually apply learning strategies, regulate learning processes, or construct mathematical learning knowledge during real classroom learning and problem solving. Future studies may use mixed-methods designs by combining questionnaires and achievement tests with interviews, classroom observations, think-aloud protocols, learning journals, or problem-solving records. Such methods would provide deeper evidence on how mathematically gifted students develop and use MK, LK, and MLK in authentic learning contexts.

Finally, this study examined MK, LK, and MLK as predictors of mathematics achievement, but other factors may also influence mathematically gifted students’ achievement. Future research could include additional variables such as motivation, self-regulated learning, metacognition, mathematics anxiety, family support, classroom environment, teacher support, and learning opportunities. Future studies may also use confirmatory factor analysis or structural equation modeling to further validate the proposed MK-LK-MLK framework and examine the direct and indirect relationships among these variables.

Overall, future research should expand the sample, improve the identification of mathematically gifted students, use broader and more diverse research methods, and further test the theoretical framework proposed in this study. Such efforts would provide a more comprehensive understanding of how mathematically gifted students develop mathematical learning knowledge structures and how these structures contribute to mathematics achievement.

## Data Availability

The datasets presented in this article are not readily available because they contain information from minor participants and are subject to privacy and ethical restrictions. Requests to access the datasets should be directed to Wei Zhou, 3027342554@qq.com.
